# The evolutionary dynamics of how languages signal who does what to whom

**DOI:** 10.1038/s41598-024-51542-5

**Published:** 2024-03-27

**Authors:** Olena Shcherbakova, Damián E. Blasi, Volker Gast, Hedvig Skirgård, Russell D. Gray, Simon J. Greenhill

**Affiliations:** 1 Department of Linguistic and Cultural Evolution, Max Planck Institute for Evolutionary Anthropology, 04103 Leipzig, Germany; 2 Catalan Institute for Research and Advanced Studies (ICREA), Barcelona, 08010 Spain; 3 Center for Brain and Cognition, Pompeu Fabra University, Barcelona, 08018 Spain; 4 Department of English and American Studies, Friedrich-Schiller University of Jena, 07745 Jena, Germany; 5 School of Psychology, University of Auckland, 1010 Auckland, New Zealand; 6 School of Biological Sciences, University of Auckland, 1010 Auckland, New Zealand

**Keywords:** Anthropology, Cultural evolution

## Abstract

Languages vary in how they signal “who does what to whom”. Three main strategies to indicate the participant roles of “who” and “whom” are case, verbal indexing, and rigid word order. Languages that disambiguate these roles with case tend to have either verb-final or flexible word order. Most previous studies that found these patterns used limited language samples and overlooked the causal mechanisms that could jointly explain the association between all three features. Here we analyze grammatical data from a Grambank sample of 1705 languages with phylogenetic causal graph methods. Our results corroborate the claims that verb-final word order generally gives rise to case and, strikingly, establish that case tends to lead to the development of flexible word order. The combination of novel statistical methods and the Grambank database provides a model for the rigorous testing of causal claims about the factors that shape patterns of linguistic diversity.

## Introduction

The main goal of recent research in comparative linguistics has been to document and explain linguistic diversity^1–4^. One major dimension of cross-linguistic variation is the way in which languages use grammatical means to mark core arguments. In other words, how do languages signal "who does what to whom". Most languages mark this relationship overtly in some fashion, but they differ in the choice of grammatical means. In utterances with an Agent (A) and a Patient (P), for example, *Henry kissed Mark*, languages need to signal which argument maps onto which role. To highlight the distinct roles of these two arguments, languages choose between grammatically marking verbs or nouns, enforcing rigid word order, or relying on semantic cues, such as animacy. Standard English typically differentiates between A (the more agent-like argument in a transitive clause) and P (the more patient-like argument in a transitive clause) arguments by rigid word order. For example, in the sentence *Cats chase mice* only the order of the arguments—more active A followed by more passive P—defines who chases whom. If the word order was flipped, *Mice chase cats,* the roles would also be reversed. In another sentence—*The boy visits the grandparents—*an index *-s* of the A argument is attached to the verb to signal who does what to whom. It is not possible for the *grandparents* to be the A-argument (‘who’), otherwise the verb *visit* would not have the *-s* suffix. In contrast, other languages assign two different forms to A and P arguments themselves either by different roots or by morphology. This is known as case marking. For instance, in the sentence in the Mongolian language Ordos, *cinggis kaan cimbulama-ig jalaba* ‘Chinggis Khan invited Chimbu Lama’^5^, the suffix *-ig* marks the P argument—making clear that Chimbu Lama is the Patient—the invitee. Conversely, in the Papuan language Bauzi the A arguments are marked with *-at* and the P arguments are not marked. For example, in this sentence: *Dam-at elae ote* ‘People killed brother-in-law’^6^, the word *Dam* (people) carries the A-marker *at* which indicates that it is the Agent of this clause. Out of these strategies, case and word order have received the most attention in the literature (around 79% of languages in our sample have either of the strategies) and will be the focus of this paper.

It has been argued that the means for marking core arguments do not vary randomly across languages but rather the preferred strategy of argument marking in a particular language appears to be connected to word order. First, if a language has verb-final word order, it tends to exhibit case^7–11^. Conversely, verb-medial languages are expected to be caseless^12^. Second, if a language has flexible word order, it is also likely to have case^13–16^, which is equivalent to the alternative conceptualization of the same hypothesis as case being absent in rigid word order languages^12^. Flexible word order, unlike rigid word order, implies that the positions of A and P arguments can vary without changing the meaning of the sentence. These two hypotheses have arisen independently in different studies and, to date, the interaction between these hypotheses has been largely overlooked (but see^12,17^). A major challenge lies in uncovering the causality behind the occurrence of case in both verb-final and flexible word order languages.

There are different hypotheses that attempt to explain these two grammatical patterns. According to the *processing hypothesis,* the association between case marking and verb-final word order satisfies our processing preferences in that grammatical relations can be identified early in the sentence^9,18,19^. Hence, these studies point to three complementary tendencies in marking core arguments at the beginning of the sentence: (1) verb-final languages mark case on nominal words; (2) verb-initial languages index both A and P arguments on verbs; and (3) verb-medial languages make use of rigid word order more than the two mentioned word order type languages^9,10^.

Another explanation stems from the *noisy-channel hypothesis*^11,20^: when A and P arguments are adjacent as in verb-final languages, the chances for misinterpreting the roles of the two are higher than when the arguments are separated by a verb. Specifically, verb-medial languages are more robust to noise disrupting the linguistic signal because when one of the arguments cannot be recovered, the position of the available argument on either side of the verb will inform its role (A or P), which is not the case in verb-final languages with both arguments preceding the verb. Thus, explicit disambiguation between two arguments in the form of case marking occurs more frequently in verb-final languages that are less robust to noise than verb-medial ones^7,9,10^.

The co-occurrence of flexible word order and case is explained by the *trade-off hypothesis*^14^, which claims that languages should generally aim for a balance between clarity and ease of production. Redundancy would manifest itself in using several means for the same purpose simultaneously. This could be advantageous for the recipient as it would provide a more robust signal if there is noise or misunderstandings but can be costly for the sender. The economy principle on the other hand dictates that the sender should use as few resources as possible to get the message across, avoiding redundancy and unnecessary content. Both rigid word order and case are two strategies used to signal “who does what to whom”. To avoid redundancy and be economic, languages should either have case marking and flexible word order, or no case marking and rigid word order^14^. As corpus studies show, more flexibility in the word order is indeed associated with the presence of case^15,16^. This would imply that languages with rigid word order and case marking should lose one of the strategies, whereas languages with flexible word order and no case marking should acquire one of them.

In contrast, the relationships between these features could also be asymmetric: languages lacking case are likely to have rigid word order, but languages with case do not necessarily allow the flexible ordering of core arguments^13^. According to a theory of *licensing and structural case*^13^, rigid word order can replace case as a strategy for discriminating between core arguments after the loss of morphology, but languages with case often rely on rigid word order as the second strategy. This view was corroborated by corpus studies^12,15,16^.

Synchronic distributions of grammatical features are claimed to have diachronic (historical) explanations behind them^21–24^. In other words, for certain feature combinations to currently have a universal character^3,7,23^, two of the following scenarios must have been in play in the past. Either some languages from distinct language families and locations must have been preserving the inherent combination of the given features. Or, alternatively, languages lacking those features must have been undergoing changes to achieve this preferred state. From a diachronic perspective, the co-occurrence of case with either verb-final or flexible word order should result from a process where languages are more prone to possess both or neither of these combinations, and languages with one of the features will rapidly evolve to gain the other or lose the existing one.

Below we summarize the hypotheses and results of corpus studies linking case marking to verb-final or flexible word order and complement these with matching diachronic patterns (Table 1). For each of the hypotheses, we propose a diachronic scenario inferred from the previous studies that would bring about the combination of the features in question. As we can see, the implied causal relationships behind verb-final languages with case are unidirectional: verb-final word order causes the emergence of case^7,9,11,18–20^. As for flexible word order languages with case, there is less certainty about the order of changes behind this pattern. The presence of case causing the shift in word order towards flexible word order has been claimed to be likely^14^ and unlikely^12,13,15,16^. In addition, we observe the reverse causal relationship of flexible word order languages gaining case^12,14–16^. Furthermore, the lack of case was also thought to trigger the loss of flexible word order, leading to caseless languages with rigid word order^13^.

**Table 1:** Representation of diachronic scenarios underlying the interaction between case and word order.

Diachronic scenario	Description	Hypothesis
verb-final WO → case	verb-final word order causes case	processing hypothesis, noisy channel hypothesis, and word order universal
flexible WO → case	flexible word order causes case	trade-off hypothesis, corpus studies results
absent case → absent flexible WO	absent case causes absent flexible word order (i.e. leads to rigid word order)	a theory of licensing and structural case, corpus studies results
case → flexible WO	case causes flexible word order	trade-off hypothesis vs a theory of licensing and structural case and corpus studies results

Here we aim to infer the relationships between grammatical means of signalling “who does what to whom”. We extract information about the presence/absence of case and word order features from a global dataset of unprecedented scope—Grambank^25,26^ (see Table 2 in Materials and methods). To go beyond synchronic correlational studies, and make causal inferences, we model the evolution of these data on a new global phylogeny^27^. By inferring the order of feature changes on a phylogeny we can test claims about the causal processes underlying each of the scenarios. To achieve this, we use methods derived from computational phylogenetics. The numerous parallels between biological evolution and linguistic change mean that these tools are increasingly being used in historical linguistics^28–30^. Importantly, these methods tackle the problem of phylogenetic non-independence, or Galton’s problem^31^. This cannot be resolved in a sufficient way by sampling languages from different families since more closely related language families will still be more prone to have common features than two more distant families^28,32^. Not accounting for this violates the assumption of data points being independent from each other, which is imposed by many statistical tests. By contrast, evolutionary methods used here take into consideration the genealogical relationships between languages and offer a nuanced view on all possible language change patterns for a given feature pair rather than illustrating frequencies of feature occurrence. First, we test whether the traits pattern strongly on the phylogeny^33,34^ by measuring phylogenetic signal (i.e. similarity due to inheritance). We measure the phylogenetic signal of case, verb-final and flexible word order using the D metric^35^. Features with high phylogenetic signal are shared by closely related languages that inherited the values from their ancestors, and the variation within such features is largely explained by phylogenetic relationships between languages rather than the influence of other predictors. Conversely, the values of the features with low phylogenetic signal differ greatly between close relatives^35^, which can imply that these features have been subject to change or areal diffusion. Next, since significant phylogenetic signal indicates that the distribution of all three variables has been constrained by evolutionary history, we apply a phylogenetic path analysis^36,37^ that allows us to evaluate competing causal models while controlling for phylogenetic autocorrelation and establish how the different tactics for signaling “who does what to whom” have emerged. The phylogenetic path analysis allows us to control for phylogenetic autocorrelation in a powerful causal framework but cannot resolve the potential confounding effects of spatial proximity. This analysis is based on phylogenetic regression, which lacks the term for residual errors not explained by phylogeny. The purpose is two-fold: 1) to assess whether phylogenetic effects alone are sufficient to explain the variation in the distribution of our grammatical features or if they need to be supplemented/substituted in the main analysis with spatial effects and 2) to establish if the results of phylogenetic path analysis are robust under a different modelling approach. We find that all three features can be modelled using only phylogenetic effects. We proceed to implement the Bayesian multilevel modelling approach in order to establish if the results of phylogenetic path analyses hold when the residual errors can be explained by phylogenetic relationships but are not limited to these.

**Table 2:** Three grammatical features that were coded for presence or absence in languages based on the values of five Grambank features.

Feature in analysis	Grambank feature value for the presence of the feature	Grambank feature
Case	Present	GB070. Are there morphological cases for non-pronominal core arguments (i.e. S/A/P)?
Verb-final word order	Present	GB133. Is a pragmatically unmarked constituent order verb-final for transitive clauses?
	Absent	GB131. Is a pragmatically unmarked constituent order verb-initial for transitive clauses?
	Absent	GB132. Is a pragmatically unmarked constituent order verb-medial for transitive clauses?
Flexible word order	Absent	GB136. Is the order of core argument (i.e. S/A/P) constituents fixed?

## Results

### Phylogenetic signal

We measure the fit to the phylogeny for the three grammatical features by estimating their *D* values^35,38^. All three grammatical features show significant patterning with the phylogeny (probability of *D* difference from 1.0): case (*D* = 0.34, p < 0.01), flexible word order (*D* = 0.74, p < 0.01), and verb-final word order (D = 0.12, p < 0.01). This suggests that the distribution of these variables across languages is moderately to strongly driven by historical inheritance between related languages. In addition, verb-final word order (*D* = 0.12, probability of difference from 0 is non-significant) is not significantly different from 0, which indicates that it is evolving in a Brownian manner on the tree consistent with very strong phylogenetic coupling. Therefore, all *D* values indicate that tests involving all three of the variables need to control for phylogenetic dependency.

### Phylogenetic path analysis

**Figure 1:**
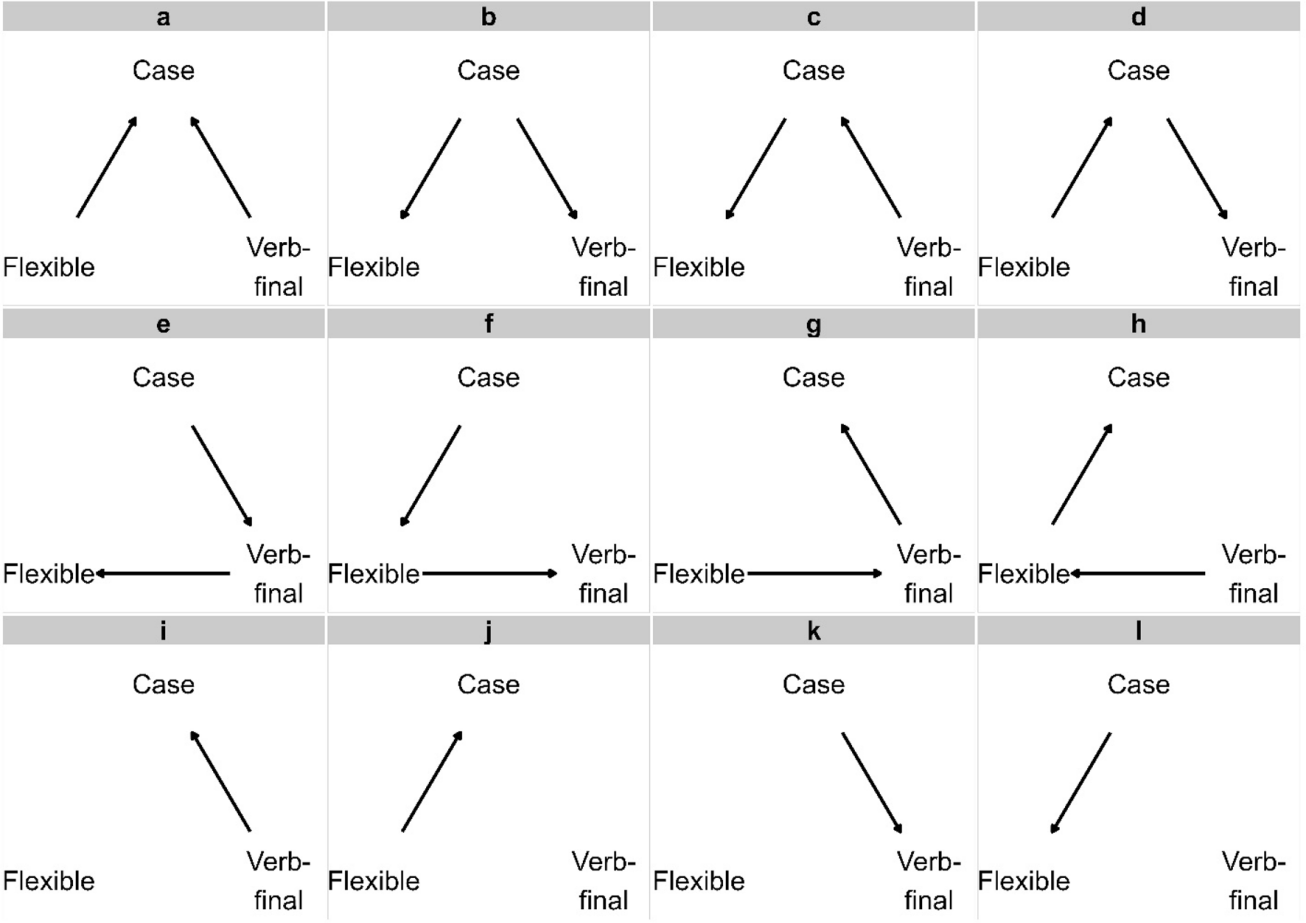
12 competing causal models representing the potential relationships between case and two word order features, verb-final word order and flexible word order. Model *i* represents the diachronic scenarios inferred from processing hypothesis, noisy-channel hypothesis, and the word order universal. Model *j* stems from a theory of licensing and structural case and the results of empirical corpus studies. Other models represent the inverted directions of these models (*k* and *l*) and the possible combinations of causal paths between three or two features (*a*-*h*) that additionally test for the potential indirect relationship of one of word order features on case.

Phylogenetic path analysis^36,37^ within *phylopath R* package^39^ allows to control for phylogenetic non-independence by incorporating a phylogeny and helps to evaluate and compare multiple causal models that include several variables. To infer the direction of causality between case marking and two word order features, we test the causal models that represent the discussed diachronic patterns as well as possible conditions that were unaccounted for by previous studies. For instance, we test not only whether verb-final word order causes case as was suggested in prior literature, but also the reverse path where case triggers the shift to verb-final word order. Additionally, we explore the scenarios where two word order features interact with each other, which has not been previously explored. Our comprehensive model set captures the conditions where word order leads to the development of case, case leads to the development of word order and where one of the three features acts as both a cause and an effect with regard to two other features. This model set also includes four simple models with one causal path. As a result, we test 12 causal models in total (see Fig. 1).

**Figure 2:**
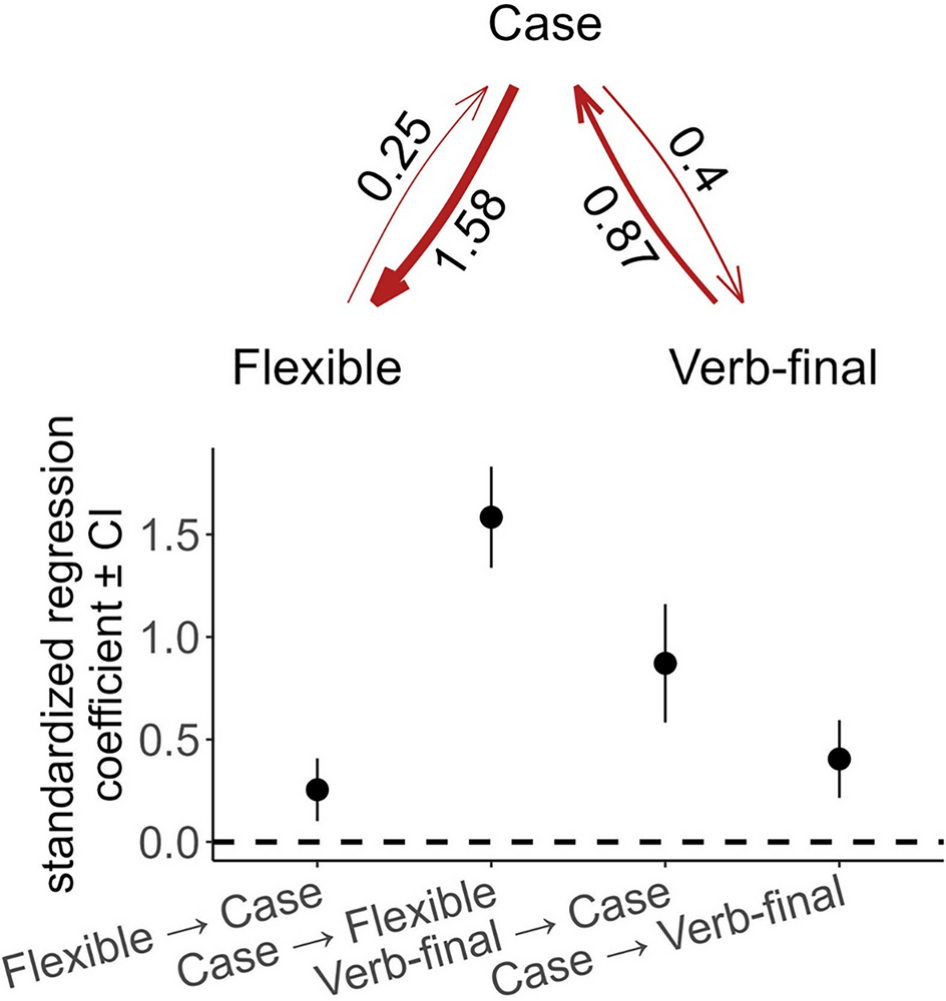
The averaged standardized regression coefficients (ranging from 0.25 to 1.58) of the best-fitting models with their Confidence Intervals. The coefficient values range from 0.1 to 0.41 for Flexible → Case, from 1.34 to 1.83 for Case → Flexible, from 0.58 to 1.16 for Verb-final → Case, and from 0.24 to 0.59 for Case → Verb-final, where values indicate how strongly the features are correlated. All identified causal links are positive and robust.

Out of 12 tested models, the top-ranked causal models are *c* (Verb-final → Case → Flexible word order) (CICc weight = 0.56), *b* (Case → Flexible word order, Case → Verb-final word order) (CICc weight = 0.21), and *d* (Flexible word order → Case → Verb-final word order) (CICc weight = 0.21). We use conditional model averaging to obtain the averaged coefficients of these best-fitting models and summarize the resulting average model in Fig. 2. The positive standardized regression coefficients show that case and word order features reinforce each other rather than leading to their loss. Verb-final languages are more likely to gain case, and where case is available, languages are more likely to develop flexible ordering of core arguments. The reciprocal paths are also possible but are not as common. The shift to verb-final word order in languages with case is two times less likely than a verb-final language shifting to case. Similarly, the emergence of case in flexible word order languages is six times less likely than the development of flexible word order in languages with case. The rest of the models have larger C-statistic information criteria corrected for small sample sizes (CICc) (∆CICs > 2), and are rejected based on the available evidence (*p* values < 0.05 indicate rejection of the model) (see Table 3 in Methods and materials).

**Table 3:** The results of the comparison of fitted causal models in phylogenetic path analysis.

model	CICc	∆CICc	relative likelihood	CICc weight	n of independence claims	n of parameters	C statistic	p-value
**c**	**13.64**	**0**	**1**	**0.56**	**1**	**5**	**3.6**	**0.17**
**b**	**15.62**	**1.98**	**0.37**	**0.21**	**1**	**5**	**5.58**	**0.06**
**d**	**15.62**	**1.98**	**0.37**	**0.21**	**1**	**5**	**5.58**	**0.06**
g	20.31	6.67	0.04	0.02	1	5	10.27	0.01
a	21.54	7.9	0.02	0.01	1	5	11.5	0
k	27.22	13.59	0	0	2	4	19.2	0
i	29.8	16.16	0	0	2	4	21.77	0
f	29.9	16.26	0	0	1	5	19.86	0
e	48.59	34.96	0	0	1	5	38.56	0
l	52.71	39.07	0	0	2	4	44.68	0
h	55.96	42.32	0	0	1	5	45.92	0
j	65.44	51.81	0	0	2	4	57.42	0
null	70.23	56.6	0	0	3	3	64.22	0

Three top models in bold have the lowest CICc (the difference between CICc of the top model *c* and two runner-up models is less than 2) and non-significant C-statistic, which indicates that all three models describe supported causal scenarios.

### Bayesian multilevel modelling

**Figure 3:**
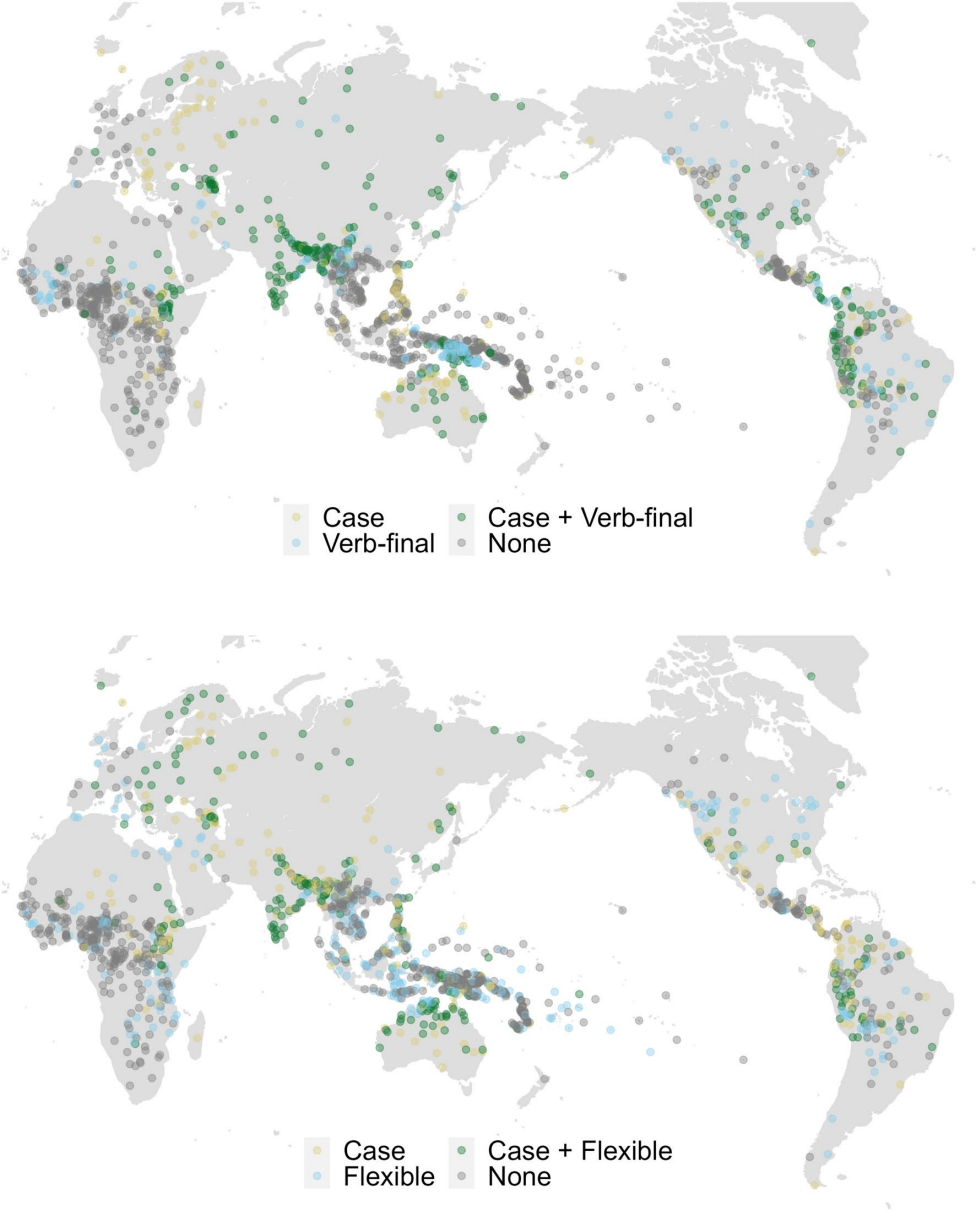
Maps showing the distribution of case and word order patterns in the language of the world (represented by colored dots); yellow: case without the word order type indicated in the map (11% of languages without verb-final and 17% of languages without flexible word order); blue: word order type indicated in the map without case (15% of verb-final languages and 22% of flexible word order languages); green: presence of case and word order type indicated in the map (22% of languages have case and verb-final word order and 16% of languages have case and flexible word order); gray: absence of case and word order type indicated in the map (in 52% of languages verb-final and case are absent, and in 46% flexible word order and case are absent). The top map depicts the combination of case with verb-final word order, while the bottom map illustrates case with flexible word order. The Indian subcontinent and the Caucasus region contain languages that almost exclusively combine case and verb-final word order. Many languages that combine case and flexible word order are located in South America, Eurasia, the Indian subcontinent, and Australia.

**Figure 4:**
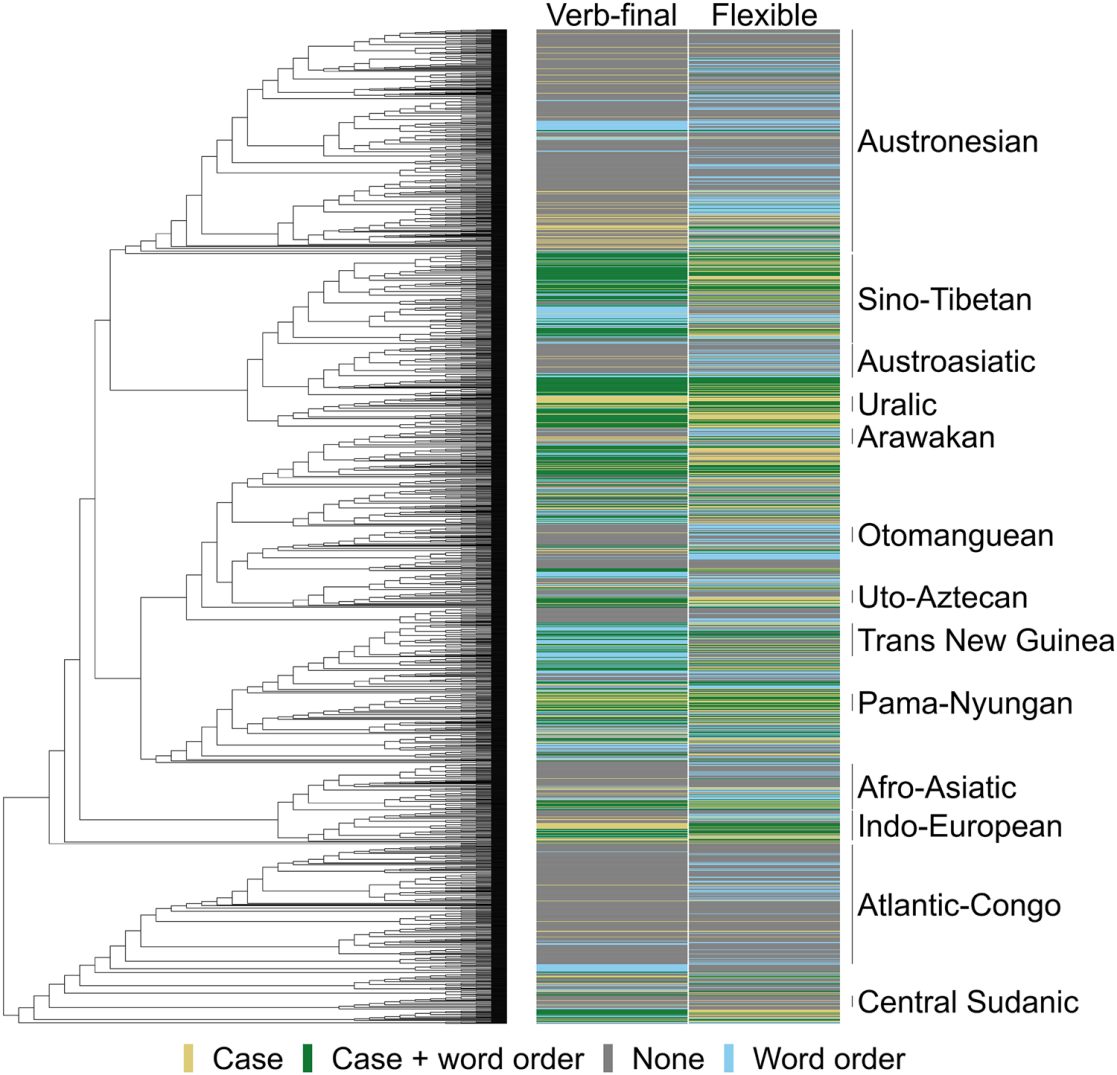
Phylogeny (global tree on the left) and the distribution of case and word order patterns (colored blocks on the right); yellow: case without the word order type indicated in the column header; blue: word order type indicated in the column header without case; green: presence of case and word order type indicated in the column header; gray: absence of case and word order type indicated in the column header. Verb-final word order is a more stable feature than flexible word order, which is visible from larger blocks of color sequences across languages representing its presence (blue and green) or absence (yellow and gray). By contrast, flexible word order can be present or absent in groups of closely related languages.

The languages in our large sample are not independent from each other due to shared inheritance and/or spatial proximity (see Fig. 3 for geographic distribution of case and word order features in isolation and combination with each other and Fig. 4 for their distribution on the phylogenetic tree). When this non-independence is not accounted for, it may lead to spurious correlations between our grammatical features of interest. While phylogenetic non-independence has been accounted for in the phylogenetic path analysis, the compared causal models do not explicitly control for the effects of spatial proximity. Even though the global tree uses geographic information to resolve the position of uncertain branches in the tree and thus contains an areal signal, it is not clear if incorporating spatial information directly could enhance our understanding of how case and word order features are distributed. To address this, we assess whether phylogenetic effects alone are sufficient to explain the variation in the distribution of our grammatical features or if they need to be supplemented/substituted in the main analysis with spatial effects. The results below are in line with the strength of phylogenetic signal: as was previously shown, the distribution of flexible word order fit the phylogeny worse than two other variables, and this variable is the only one that is best predicted by either spatial random effects or the combination of spatial and phylogenetic effects.

As the first step, we test whether the distribution of case marking and word order features is driven by 1) phylogenetic similarity, 2) spatial proximity, or 3) both of these sources of independence in a logistic regression model implemented with Bayesian multilevel modelling in *brms*^40^ in *R*^41^. We use K-fold cross-validation with the conventional 10 subsets as recommended in^42^ to compare our three sets of models with two random effects (phylogenetic relationships and spatial autocorrelation) in isolation and in combination. Model comparison for all three variables reveals phylogenetic-only models to be equivalent with spatial-only and/or spatiophylogenetic models in their predictive performance^43^ (see Table S1 in Supplementary Materials). This means that these variables can be modelled using phylogenetic random effects alone, and there is no evidence that adding an explicit spatial element would explain this variable above and beyond the phylogeny.

Next, to establish whether the phylogenetic path analyses results arrived at with phylogenetic regression remain robust using Bayesian mixed modelling approach, we fit the best-supported *b*, *c*, and *d* models in *brms* and check whether the previously reported relationships hold (see Table S2 in Supplementary Materials). We choose to model all variables with phylogenetic effects, given the lack of substantial model improvement when including spatial effects, and to ensure better comparability of the results from both methods. We find no evidence for the effects of flexible word order on case (model *b* and *c*) and case on flexible word order (model *d*) under this approach.

 We also observe higher coefficients between verb-final word order and case in the results of Bayesian multilevel modelling than in the phylogenetic path analysis model. Additionally, in contrast to phylogenetic path analysis, the coefficient for the path from verb-final to case (1.49, 95% credible interval 0.86–2.15 in model *c*) is lower than the coefficient of the opposite effect (1.76 with 95% credible interval 0.67–3.01 in model *b* and 1.75 with 95% credible interval 0.66–2.99 in model *d*). The wide credible intervals of the latter path encompass all values of the credible intervals of the opposite path. This indicates that these results imply a higher degree of uncertainty in the unidirectionality of the relationship between these two variables compared to phylogenetic path analysis.

## Discussion

This study demonstrates how the combination of large linguistic datasets and new computation methods can be used to answer long-standing questions about the causal pathways that have shaped linguistic diversity. In line with previous studies^7–11,13–16^, we find strong support for the co-occurrence of case with (1) verb-final word order (rather than non-verb-final word order) and (2) flexible word order (rather than rigid word order). Given the robust support for this synchronic pattern, the natural question that arises concerns how this pattern emerges diachronically. The combinations of case marking with the two word order characteristics, or their avoidance, could have arisen either due to the stability of the feature combinations or multiple systematic changes that resulted in the currently widespread combinations of features.

We confirm two tendencies of the presence of case in verb-final and flexible word order languages compared to languages with other word order features. The causal relationships and mechanisms behind these two patterns are not the same. Case is generally set in motion by verb-final word order due to the processing preferences in favor of this feature combination and low robustness of verb-final languages in the absence of case. The presence of case tends to trigger the shift from rigid to flexible ordering of core arguments, which avoids redundancy and enables flexible word order to signal whether core arguments represent given or new information. The reciprocal paths are also attested but less likely than the dominant ones.

Changes towards co-occurrence and co-absence of both features have been prominent in the history of languages in our sample. A family that illustrates the interaction of both stability and change in the studied features is Indo-European. Case on core arguments is a feature inherited by some Indo-European languages, along with verb-final (in the Indic branch) or flexible word order (in the Slavic branch). Other languages—for instance, from the Italic and Germanic branches, failed to preserve case and underwent word order changes^17^. Thus, Indo-European languages exemplify both retention of a preferred pattern (case in combination with flexible or verb-final word order), and change towards the absence of both features (see Figure S3 in Supplementary Material). Similar scenarios are also found in Sino-Tibetan and Uto-Aztecan languages. For instance, unlike Northern Uto-Aztecan languages, many Southern Uto-Aztecan languages failed to retain verb-final word order and case (see Figure S4 in Supplementary Material). Similarly, in the Karenic branch of Sino-Tibetan case is absent and the shift in word order from verb-final to verb-medial word order took place, whereas many other Sino-Tibetan languages preserve both features (see Figure S5 in Supplementary Material). The Macro-Tani branch of the Sino-Tibetan family might exemplify another change pattern: the shift away from rigid to flexible word order in languages with case. Most languages from this branch possess case and some, such as Nyishi-Hill Miri, Galo, and Milang, must have gained flexible word order (see Figure S6 in Supplementary Material).

The best-fitting causal models suggest that a) languages with verb-final word order are inclined to gain case on core arguments than languages with non-verb-final order and b) languages where case is available tend to shift their word order to allow flexible ordering of core arguments as opposed to caseless languages. The finding that verb-final word order leads to the presence of case (verb-final word order → case) might result from the combined effect of (1) processing preference for establishing grammatical relations early in the sentence^9,18,19^, (2) sensitivity of verb-final languages to the noise in the linguistic signal^11,20^, and (3) adhering to a word order universal^7^. While the previous research suggests that the relationship between verb-final word order and case marking is unidirectional, we show that both directions are possible, but the development of case in verb-final languages is more likely than the reverse scenario. This finding is in line with one of the diachronic scenarios behind the noisy-channel hypothesis, namely, that verb-final languages develop case to maximize meaning recoverability of semantically reversible events even under noise conditions^11^. The alternative diachronic scenario of the noisy-channel hypothesis about caseless verb-final being likely to shift to verb-medial order has not been tested here. Additionally, there might be biases towards developing/maintaining both of these features independently, so that the combination of verb-final languages with case are common. For instance, verb-final word order has been suggested as the order of the ancestral language^44^ and the default/preferred order in human communication^11,20,45^. These accounts are primarily grounded in the verb-final gesturing bias for communicating semantically reversible events, which was found in the participants with native languages differing in the position of the verb. The grammaticalization of case might be a common strategy for marking “who does what to whom” due to (1) its explicitness compared to rigid word order, which allows for robust identification of core arguments when communication is affected by noise^11^, and (2) its higher potential for disambiguation of two arguments compared to verbal indexing^46^.

Conversely, prior studies do not agree on the directionality of the relationship between case and flexible word order. The trade-off hypothesis suggests that reciprocal paths between these features should be possible as they prevent both economy and redundancy. While we find support for both directions in the phylogenetic path analysis, the strongest path in our analysis (case → flexible word order) is much more common than the reciprocal path (flexible word order → case). These relationships deserve more attention in the future studies since they were not confirmed under the Bayesian multilevel modelling approach. In contrast to our phylogenetic path analyses results, some studies argue that the reciprocal path is the primary route, and deny the emergence of flexible word order from case^12,13,15,16^. This view has been motivated by abundant examples of languages retaining rigid word order despite already disambiguating core arguments with the help of case. This pattern is encountered, for instance, in Faroese and Standard Arabic, both of which possess case and maintain rigid word order.

Below we consider the reasons that explain why case-making languages might resist the pressure to allow flexible ordering of core arguments. Firstly, some languages might be changing at a slower pace than others and thus currently show no observable changes in their word order structure. For instance, the speakers of Faroese are geographically isolated from the speakers of other languages, which might be responsible for overall fewer changes in the Faroese grammatical system when compared to Norwegian, which underwent more contact with other languages, but fewer changes compared to even more isolated Icelandic^47,48^. As for Standard Arabic, unlike most spoken Arabic varieties that have lost case marking, it might have retained the current case system and resisted word order changes because of its overt prestige, high variety function in the diglossic communities of Arabic speakers, and the pressure for standardization.

Secondly, the combination of case and rigid word order can reflect accretion of redundancy in the core argument marking (two strategies instead of one) and the tendency of languages to develop more grammatically complex phenomena over time^49^. Some languages seem to favor using multiple strategies for keeping arguments apart over failing to disambiguate core arguments. This is especially evident taking into account that the third strategy of core argument marking—verbal indexing—has not been found to be in complementary distribution with two other strategies on a cross-linguistic sample^50^. Similarly, there appears to be no relationship between verbal indexing and case marking in many Papuan languages^51^.

Thirdly, the relationship between case and word order (rigid vs flexible) might be more challenging to disentangle than it appears. Our study corroborates the global complementary distribution of case marking and rigid word order. However, their functions do not overlap entirely. Specifically, both strategies can be retained when the function of case goes beyond reinforcing distinctions between A and P made by rigid word order. For instance, apart from marking “who does what to whom”, case can provide additional semantic/pragmatic information about core arguments, such as (in)animateness or (in)definiteness and whether they represent given or new information^52^. Importantly, signaling whether A and P represent given or new information can be also done in languages with flexible word order^46^. For instance, in Finnish, the pragmatically unmarked order is verb-medial, with A arguments preceding and P arguments following the verb. However, the reversal in A and P arguments positions (P argument precedes the verb which is then followed by A argument) indicates that the P argument represents new information whereas the A argument is known or given information^53^. The fact that case and ordering of core arguments enable not only argument disambiguation, but also marking distinctions in information structure, can be the key to understanding why some languages respond to the pressure to possess either case or rigid word order whereas others maintain both.

Apart from the changes in case and word order features, the preferred patterns of discussed feature co-occurrences can prevail due to their retention across families. For instance, the Austronesian, Austroasiatic, Tai-Kadai, Central Sudanic, Mayan, and Atlantic-Congo families display considerable stability. They generally lack case and these two word order features (see Figure S1 in Supplementary Material). The opposite pattern, the presence of all three features, is evident in most Dravidian languages. Another example of stability is the presence of case marking that is shared by many Uralic languages. In all branches but Finnic, case marking is accompanied by verb-final and/or flexible word order, supporting the pattern of the examined co-occurrences. Similarly, most Nakh-Daghestanian, Ta-Ne-Omotic, and Turkic languages have both case and verb-final word order (see Figure S2 in Supplementary Material). Overall, verb-final word order shows a strong phylogenetic signal on the global tree, especially compared to flexible word order. Taken together, our results imply that common descent and resistance to changes against the preferred pattern are one of the factors explaining the current distribution of the feature combinations.

One limitation of our results is that our measure of word order flexibility is restricted to the order of the core arguments and captures the binary distinction between rigid and flexible word order rather than a gradient taking into account variability in the positions of other elements in the clause. Future large-scale cross-linguistic studies should investigate whether a more granular interpretation of word order flexibility interacts with case in a similar way, or whether the detected effects are constrained to the flexibility in ordering core arguments.

Further work can expand on whether complementarity between morphology and syntax can also be established on a more fine-grained level^54^ and whether the changes in this domain can be systematically linked to sociolinguistic factors^50,55–57^. So far, the role of L2 speakers on case marking remains contested. For instance^56^, and^55^ find that the proportion of L2 speakers is negatively correlated with case marking, whereas^57^ finds no the relationship between the proportion of L2 speakers and case marking but reports a negative effect of population size (the total number comprises L1 and L2 speakers) on variability of core arguments order. Similarly, there is conflicting evidence on the effects of the number of L2 speakers on language structures more generally^58^, which has been suggested to be uncorrelated^59^ or even negatively correlated^60^ with morphological complexity. Future directions can also include examining the use of optional case in flexible word order languages and testing whether trade-offs are evident in how case and word order perform two functions: disambiguating between core arguments and making novel information.

## Materials and methods

### Data

To test these competing hypotheses, we obtained grammatical data from a large global dataset, Grambank^25,26^. Grambank covers information on 195 grammatical features from 2,467 language varieties. We extract the information on the presence or absence of three features: case, verb-final word order, and flexible word order of core argument constituents.

**Case:** The presence of case in our study implies the existence of a morphological distinction between core arguments marked with an affix, clitic, tone, vowel alternation or lengthening. While Grambank codes the presence of case on any core argument (S (Subject of intransitive verb), A or P), typologically, languages tend to mark the difference between A and P, if any, rather than leaving these two unmarked and having a marker on the S argument. Hence, this feature predominantly captures the presence or absence of distinctions between A and P arguments. Case also counts as present in Grambank if the marker of case is optional or if it is a portmanteau marker (e.g. not only marking P argument, but also that the noun phrase is plural). If a language has a differential subject/object marking (for instance, when only animate P arguments receive case marking), this also counts as the presence of case (for more details see the feature description in^25^).

The majority of the reviewed theories and studies focus on nouns rather than pronouns, and this is our focus here too. The reason for this is that high frequency of pronouns might help them retain the cases more successfully than nouns^14,61^, which render them less prone to be affected by the functional mechanisms discussed in the different hypotheses.

**Word order flexibility:** The notion of word order flexibility can be applied to various constructions and even to the word order of the language in general. The respective feature in Grambank addresses the presence or absence of the fixed word order of core argument constituents in languages, hence the interpretation of word order flexibility in our study is restricted to their position. This implementation differs considerably from that presented in corpus studies, which offer a more fine-grained view of the flexibility of ordering multiple elements in clauses. However, our approach can offer a valuable perspective on whether case interacts in the predicted way with interchangeable positions of the restricted set of elements: core arguments on which case marking could be used to signal “who does what to whom”. According to the Grambank coding procedure, word order is coded as flexible if the order of these core arguments and the verb can be permuted whereby i) the propositional content of the clause remains the same and ii) no intonational signaling or further elements (particles, cleft components, adjuncts) are required (see the feature page for GB136 for more information). The notion of flexible word order in this interpretation does not apply to pragmatically driven, non-canonical word orders, e.g. left- or right-dislocation, as observed in specific discourse pragmatic contexts such as topicalization, focusing, etc.^62^ (e.g. English object topicalization of the type *This, I don’t know*). The only limitation is that similar to the features addressing the presence and absence of cases, this feature targets all three core arguments—S, A, and P.

**Word-order type:** We distinguish between languages that permit only verb-final position, following both core arguments in transitive clauses, and the rest of the languages. The terms we use in relation to word order types specify only the positions of verbs, not making distinctions between the positions of A and P arguments. However, based on the bias for subjects preceding objects^20^, we can assume that the majority of languages will fall under the following groups: VSO (verb-initial), SVO (verb-medial), and SOV (verb-final). The identification of the relevant features is illustrated in Table 2.

**Phylogeny:** To model the evolutionary changes in these features over time and control for shared ancestry, we map the grammatical features onto the global EDGE (evolutionarily distinct, globally endangered) phylogeny^27^. This phylogeny is a global supertree that incorporates language classification information from Glottolog^63^ and published phylogenies, as well as their geographical locations.

### Methods

**Phylogenetic signal:** We calculated the fit to the phylogeny (phylogenetic signal) of the three variables using the metric *D*^35^ as implemented in the *phylo.d* function in the caper package^38^ in the statistical programming language R^41^. The strength of phylogenetic signal measured by the metric D ranges between two extremes: a phylogenetically random distribution with respect to phylogeny and the distribution of a maximally clumped trait. This metric quantifies phylogenetic signal in binary variables by evaluating whether they follow a Brownian evolution threshold model (*D* = 0, strong phylogenetic signal) or are distributed randomly relative to the tips of the phylogeny (*D* = 1, no phylogenetic signal). The calculated *p*-values indicate whether *D* for a particular feature is significantly different from a phylogenetically random distribution (*D* ~  = 1) or a phylogenetically structured trait (*D* ~  = 0) and those two scenarios accordingly.

**Phylogenetic Path Analysis:** To determine the associations between case marking and verb-final and flexible word order and compare the underlying causal hypotheses, we conduct the phylogenetic path analysis implemented in the R package *phylopath*^39^. Phylogenetic Path Analysis^36,37^ uses Phylogenetic Generalised Least Squares regression with the d-separation algorithm^64^ to test causal hypotheses while controlling for phylogenetic non-independence. The causal models are represented as directed acyclic graphs (DAGs), which do not allow directed cycles and latent variables^61^. This means that while the variables in our causal models might be influenced by latent sociolinguistic variables^55–57^, we cannot account for their potential influence. However, phylogenetic path analysis incorporating phylogenetic regression allows us to directly circumvent the assumption of independence of observations, which is required by most statistical tests but is violated by evolving systems like language.

Model comparison is based on examining the minimal sets of conditional independencies of the models, which will be met only for the best-fitting one(s)^65^. Conditional independencies are testable implications of the model: statements of the association between variables or the lack thereof^66^. For instance, two causal models A → B → C and B ← A → C imply distinct conditional independencies. In the first causal model, A will be associated with B, and B will be associated with C, whereas A and C will be independent from each other conditioning on B, i.e. when we know the value of B, knowing the value of A will not contribute additional information about C^66^. Under the second causal model, A and B as well as A and C are expected to be associated, while B and C will be independent conditioning on A. If one of these models underlies the distribution of data, it will prove to be the supported model, and the competitor model along with the null model of no association between three variables will be rejected (see^67^ for more details).

Model comparison in phylogenetic path analysis is based on an information criterion modified for small sample sizes (C statistic Information Criterion (CICc)^68^), which is an alternative to Fisher’s C statistic^65,69^. Both tests reflect whether the correlation structure in the data significantly differs from the expected correlation structure proposed by the tested causal model, hence the associated *p* values below 0.05 indicate unlikely causal models which should be rejected based on available evidence^36,69^. The key advantage of CICc statistic over Fisher’s C statistic is that it allows comparison between non-nested as well as nested models (i.e. those that differ from each other in having or lacking one additional path^36^).

von Hardenberg and Gonzalez-Voyer^36^ emphasize the importance of accounting for model uncertainty and the preference of model comparison over model selection. This is relevant when several of the tested models emerge as truly competitive (high *p* values > 0.05 and low differences in CICc of the model and the top model ∆CICc < 2). In such cases, a single model should not be selected over others, and model averaging should be conducted on the top models within the CICc difference of 2, which have associated non-significant p-values. We identify three models with non-significant *p* values. All of them are supported based on the CICc differences: the difference in CICc between the two runner-up models and the top model does not exceed 2 (∆CICc = 1.98) (see Table 3 for comparison between the tested models). We conduct conditional averaging to summarize the results of the top three models: the coefficients of the paths included in several models are averaged according to the CICc weights of each model, while the coefficients that occur in only one of the models remain unchanged^37^. Conditional independencies incorporated in these strongest causal models^65^ imply that verb-final and flexible word orders are independent from each other when conditioned on case. In other words, when we know the value of case, knowing the values of one word order feature will not contribute to the prediction of the other word order feature.

**Bayesian multilevel modelling**: We use Bayesian multilevel modelling implemented in *brms* ^40^ package to estimate the effects of phylogenetic relationships and spatial proximity on the probability of the presence of case marking on core arguments, verb-final word order, and flexible word order in our sample. Both random effects were included in the logistic regression models as variance–covariance matrices. The phylogenetic relationships between languages are incorporated in the model in the form of a phylogenetic variance–covariance matrix from *vcv.phylo* function in *ape* package^70^, which allows us to account for non-independence of languages due to common descent. Spatial proximity is implemented by using a spatial variance–covariance matrix estimated from the Matérn covariance function in *varcov.spatial* function in *geoR* package^71^ with the parameters *ϕ* = 1.25 (correlation function parameter) and *κ* = 1 (smoothness parameter). The idea behind this is that the covariance between the features of the languages decays with the increasing distances between them. The chosen parameters restrict this covariance to be highest between languages within distances of several hundred kilometers gradually decreasing to be unlikely beyond about 1000 km.

Since we use geographic locations of languages from Glottolog^63^ in the form of latitude and longitude (rather than polygons), the calculated distances between languages will be more accurately estimated between smaller languages and overestimated for language pairs where at least one of them is spoken across larger areas. Due to the use of the global EDGE tree^27^ that incorporates the same information on geographic locations within the phylogeographic model, the resulting phylogenetic random effects might partially absorb the effects of spatial proximity. Since our global sample includes regions with genetically diverse languages like Papua New Guinea and hence phylogenetic and spatial covariance matrices will not be identical, including the control for both sources of autocorrelation should not lead to multicollinearity in the same model.

For our three response variables, we fit three models with 1) phylogenetic random effects, 2) spatial random effects (spatial proximity), and 3) both effects (phylogenetic + spatial). The model comparison is conducted by applying K-fold cross-validation^42,72,73^ which tends to be more reliable when complex models with a large number of observations are concerned. We used K-fold cross-validation with the conventional 10 subsets as recommended in^42,43^ (see Table S1 in Supplementary Materials). To test whether the relationships proposed in the strongest models according to the phylogenetic path analysis hold when using a Bayesian approach to fit phylogenetic generalized linear mixed models, we fit the b, c, and d models using the brms package. In these models, case and verb-final word order are positively correlated, but, contrary to the phylogenetic path analysis, we find no evidence for the effects of flexible word order on case and vice versa.

### Supplementary Information

Supplementary Information.

## Data Availability

All data, code, and materials are available in the main text, the Supplementary Materials, or a public repository: https://zenodo.org/doi/10.5281/zenodo.10867981.
