# Author Correction: The evolutionary dynamics of how languages signal who does what to whom

**DOI:** 10.1038/s41598-024-67946-2

**Published:** 2024-07-31

**Authors:** Olena Shcherbakova, Damián E. Blasi, Volker Gast, Hedvig Skirgård, Russell D. Gray, Simon J. Greenhill

**Affiliations:** 1 Department of Linguistic and Cultural Evolution, Max Planck Institute for Evolutionary Anthropology, 04103 Leipzig, Germany; 2 Catalan Institute for Research and Advanced Studies (ICREA), 08010 Barcelona, Spain; 3 Center for Brain and Cognition, Pompeu Fabra University, 08018 Barcelona, Spain; 4 Department of English and American Studies, Friedrich-Schiller University of Jena, 07745 Jena, Germany; 5 School of Psychology, University of Auckland, 1010 Auckland, New Zealand; 6 School of Biological Sciences, University of Auckland, 1010 Auckland, New Zealand

Correction to: *Scientific Reports* 10.1038/s41598-024-51542-5, published online 27 March 2024

The Supplementary Information file published with this Article contained errors in Table S1 and Table S2, where the control for spatial proximity was based on the two-dimensional Euclidean distances, rather than intended great-circle distances. The original Supplementary Information file is provided below.

This error has now been corrected in the Supplementary Information file that accompanies the original Article.

### Supplementary Information

Supplementary Information.

