# Supplementary material for: The evolutionary dynamics of how languages signal who does what to whom
Source: Sci Rep. 2024 Mar 27;14:7259. doi: 10.1038/s41598-024-51542-5 (PMC10973346; doi:10.1038/s41598-024-51542-5)
Supplement: Supplementary file 1 — Supplementary Information. [file 41598_2024_51542_MOESM1_ESM.pdf]

Supplementary Materials for

The evolutionary dynamics of how languages signal who does what to whom

Olena Shcherbakova, Damián E. Blasi, Volker Gast, Hedvig Skirgård, Russell D. Gray, and Simon J. Greenhill

*Corresponding author. Email: olena_shcherbakova@eva.mpg.de

This PDF file includes:

Figs. S1 to S6
Tables S1 to S2

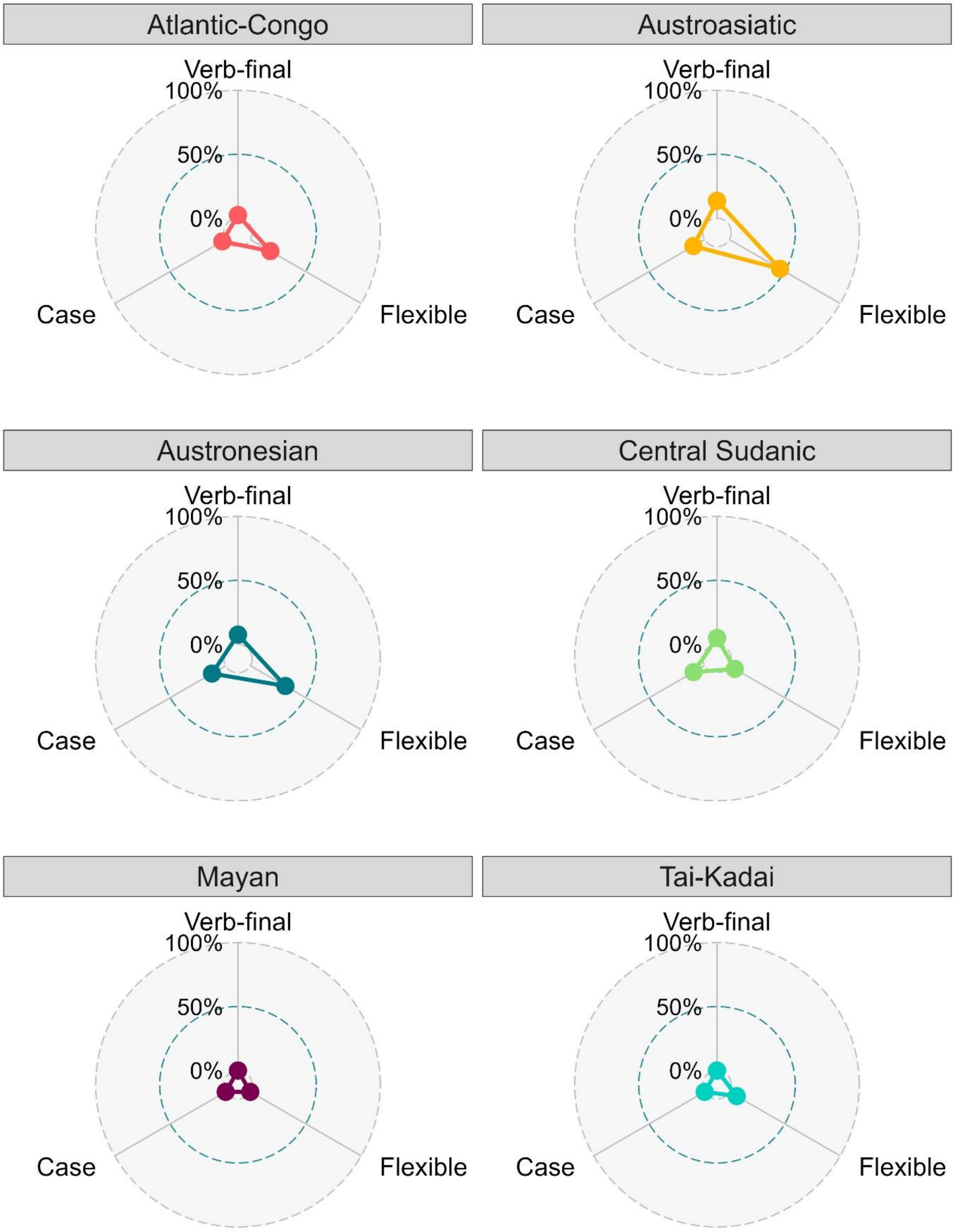

Fig. S1. The radar chart illustrating percentages of presence of three grammatical features in languages of six language families: Atlantic-Congo, Austroasiatic, Austronesian, Central Sudanic, Mayan, and Tai-Kadai. The features are predominantly absent.

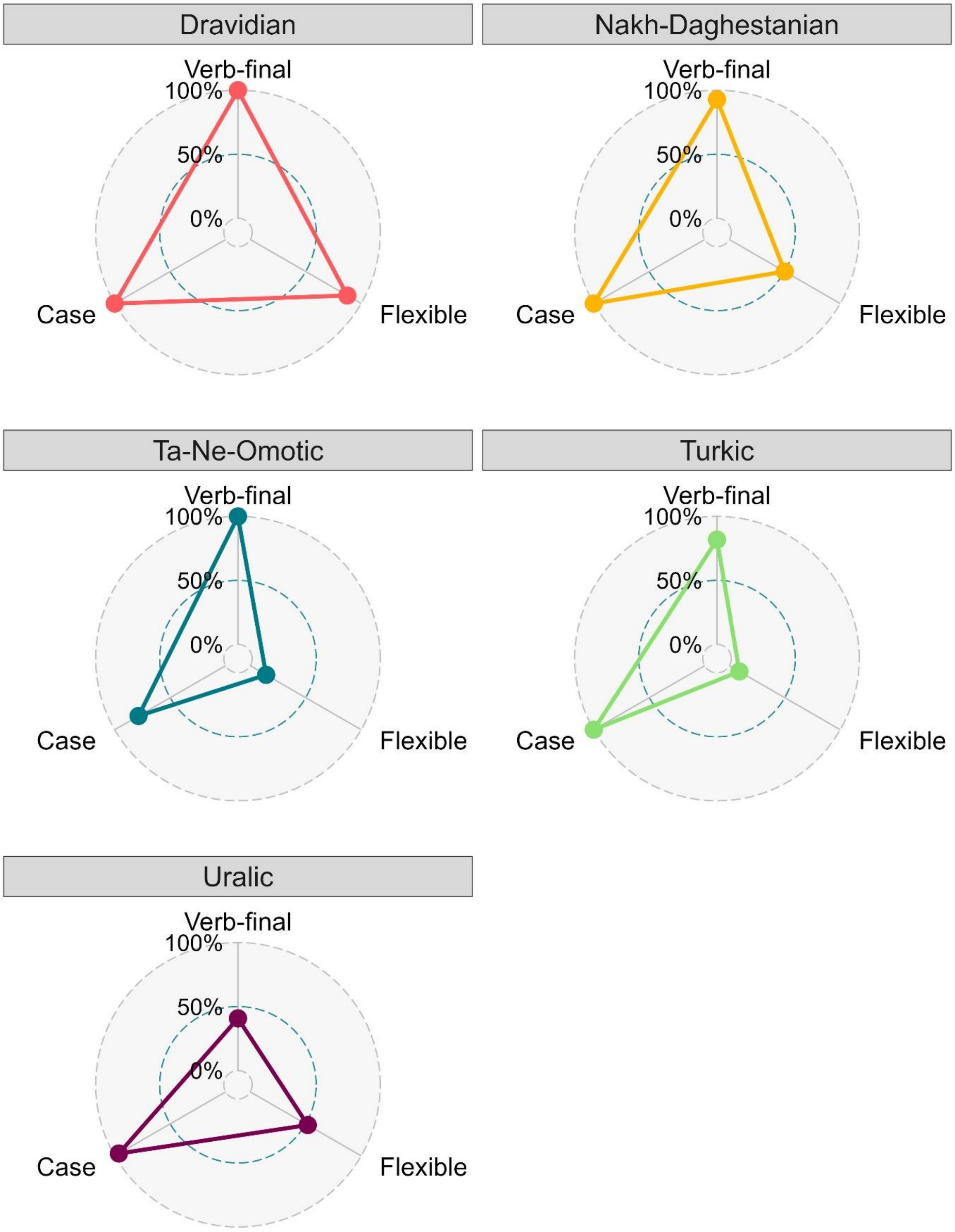

Fig. S2. The radar chart illustrating percentages of presence of three grammatical features in languages of five language families: Dravidian, Nakh-Daghestanian, Ta-Ne-Omotic, Turkic, and Uralic. In these language families, case is present at least in the majority of the languages, and verb-final word order is found in most languages of all but Uralic language families. Most

Dravidian languages and around half of Nakh-Daghestanian and Uralic languages have flexible word order.

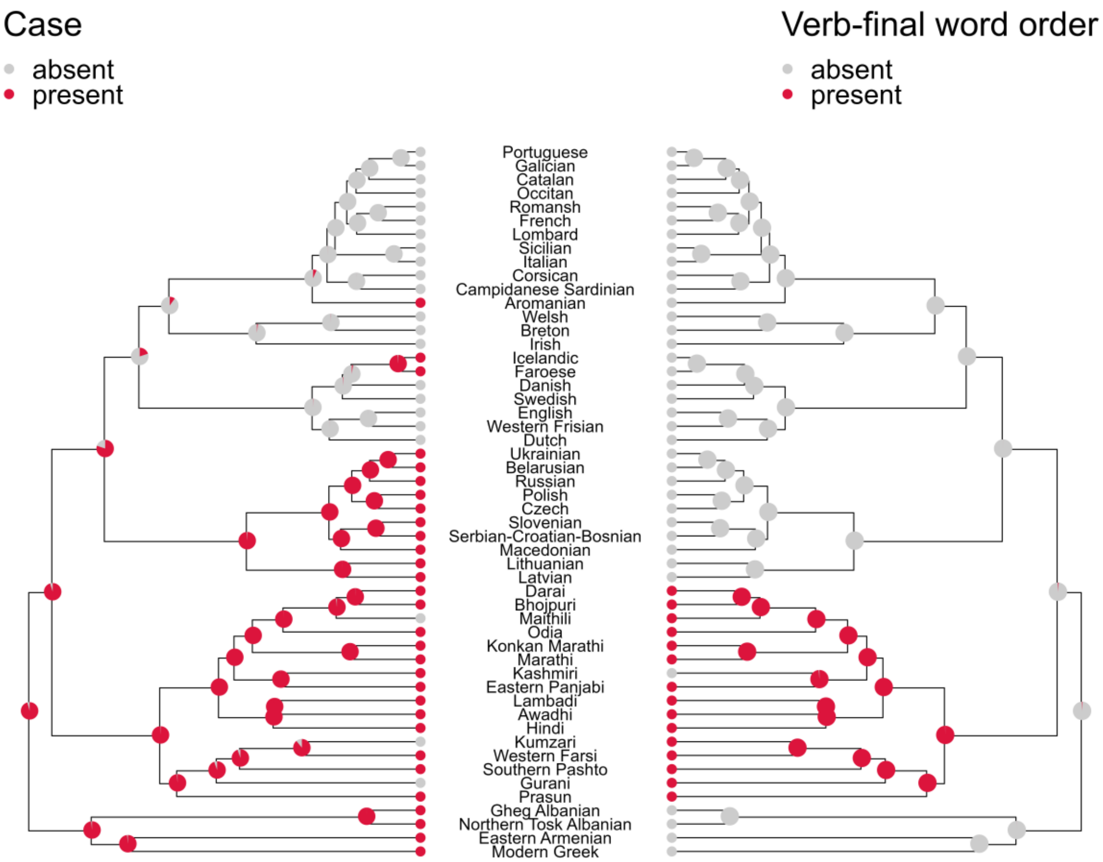
Case
absent
present
Verb-final word order
absent
present
Portuguese
Galician
Catalan
Occitan
Romansh
French
Lombard
Sicilian
Italian
Corsican
Campidanese Sardinian
Aromanian
Welsh
Breton
Irish
Icelandic
Faroese
Danish
Swedish
English
Western Frisian
Dutch
Ukrainian
Belarusian
Russian
Polish
Czech
Slovenian
Serbian-Croatian-Bosnian
Macedonian
Lithuanian
Latvian
Darai
Bhojpuri
Maithili
Odia
Konkan Marathi
Marathi
Kashmiri
Eastern Panjabi
Lambadi
Awadhi
Hindi
Kumzari
Western Farsi
Southern Pashto
Gurani
Prasun
Gheg Albanian
Northern Tosk Albanian
Eastern Armenian
Modern Greek

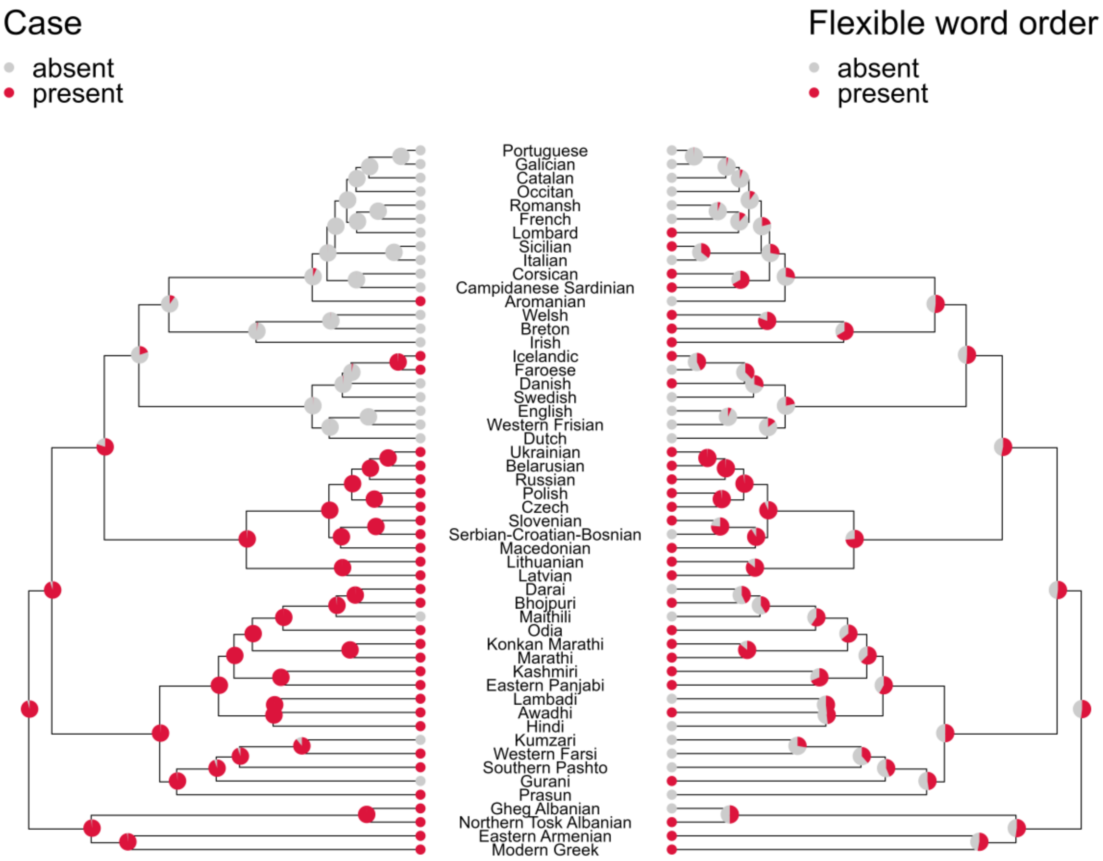
Case
absent
present
Flexible word order
absent
present
Portuguese
Galician
Catalan
Occitan
Romansh
French
Lombard
Sicilian
Italian
Corsican
Campidanese Sardinian
Aromanian
Welsh
Breton
Irish
Icelandic
Faroese
Danish
Swedish
English
Western Frisian
Dutch
Ukrainian
Belarusian
Russian
Polish
Czech
Slovenian
Serbian-Croatian-Bosnian
Macedonian
Lithuanian
Latvian
Darai
Bhojpuri
Maithili
Odia
Konkan Marathi
Marathi
Kashmiri
Eastern Panjabi
Lambadi
Awadhi
Hindi
Kumzari
Western Farsi
Southern Pashto
Gurani
Prasun
Gheg Albanian
Northern Tosk Albanian
Eastern Armenian
Modern Greek

Fig. S3. The facing trees of the Indo-European language family with values of nominal case (on the left) and word order: verb-final (top right) and flexible word order (bottom right). Red stands for the presence of the grammatical feature, while gray indicates its absence. The languages from the Indic branch preserve both case and verb-final word order, whereas many languages from the Italic and Germanic branches lost both of these features. In the Slavic branch, the presence of case was preserved along with flexible word order.

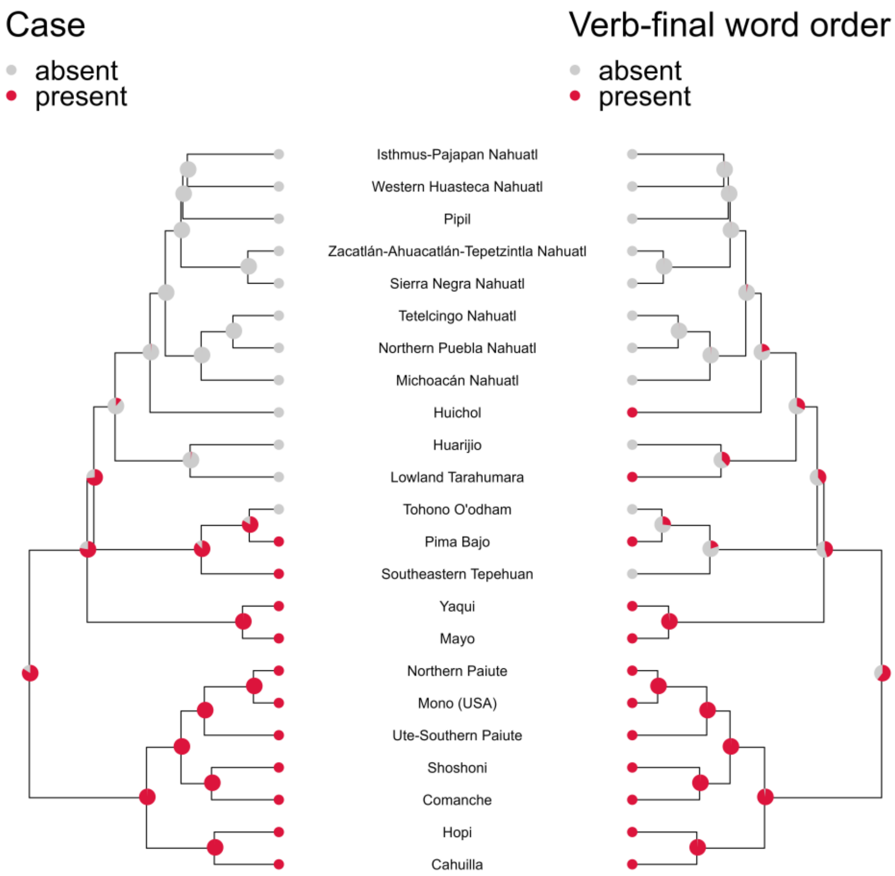
Case
absent
present
Verb-final word order
absent
present
Isthmus-Pajapan Nahuatl
Western Huasteca Nahuatl
Pipil
Zacatlán-Ahuacatlán-Tepetzintla Nahuatl
Sierra Negra Nahuatl
Tetelcingo Nahuatl
Northern Puebla Nahuatl
Michoacán Nahuatl
Huichol
Huarijio
Lowland Tarahumara
Tohono O'odham
Pima Bajo
Southeastern Tepehuan
Yaqui
Mayo
Northern Paiute
Mono (USA)
Ute-Southern Paiute
Shoshoni
Comanche
Hopi
Cahuilla

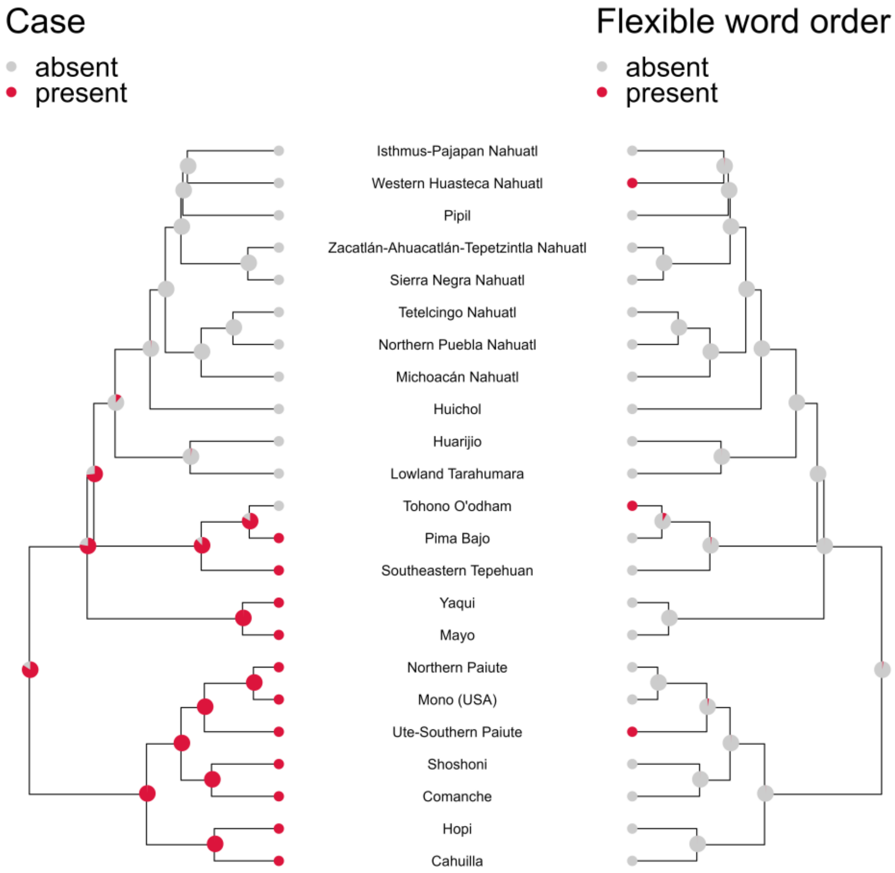
Case
absent
present
Flexible word order
absent
present
Isthmus-Pajapan Nahuatl
Western Huasteca Nahuatl
Pipil
Zacatlán-Ahuacatlán-Tepetzintla Nahuatl
Sierra Negra Nahuatl
Tetelcingo Nahuatl
Northern Puebla Nahuatl
Michoacán Nahuatl
Huichol
Huarijio
Lowland Tarahumara
Tohono O'odham
Pima Bajo
Southeastern Tepehuan
Yaqui
Mayo
Northern Paiute
Mono (USA)
Ute-Southern Paiute
Shoshoni
Comanche
Hopi
Cahuilla

Fig. S4. The facing trees of the Uto-Aztecan language family with values of nominal case (on the left) and word order: verb-final (top right) and flexible word order (bottom right). Red stands for the presence of the grammatical feature, while gray indicates its absence. Verb-final word order and case have been faithfully preserved in Northern Uto-Aztecan languages, whereas many Northern Uto-Aztecan lost both of the features.

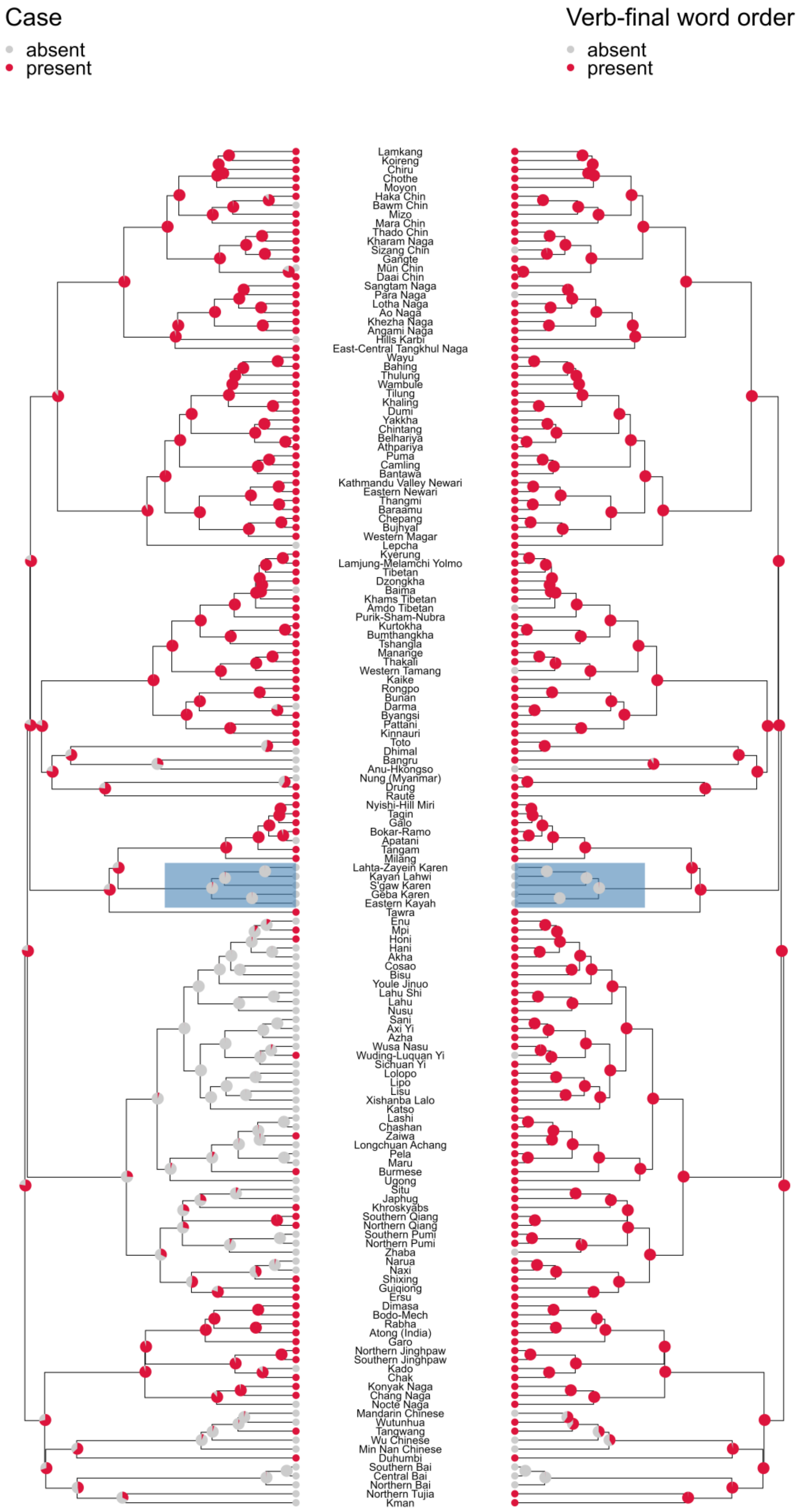

Case
absent
present
Verb-final word order
absent
present
Lamkang
Koireng
Chiru
Chothe
Moyon
Haka Chin
Bawm Chin
Mizo
Mara Chin
Thado Chin
Kharam Naga
Sizang Chin
Gangte
Mün Chin
Daai Chin
Sangtam Naga
Para Naga
Lotha Naga
Ao Naga
Khezha Naga
Angami Naga
Hills Karbi
East-Central Tangkhul Naga
Wayu
Bahing
Thulung
Wambule
Tilung
Khaling
Dumi
Yakkha
Chintang
Belhariya
Athpariya
Puma
Camling
Bantawa
Kathmandu Valley Newari
Eastern Newari
Thangmi
Baraamu
Chepang
Bujhyal
Western Magar
Lepcha
Kyerung
Lamjung-Melamchi Yolmo
Tibetan
Dzongkha
Baima
Khams Tibetan
Amdo Tibetan
Purik-Sham-Nubra
Kurtokha
Bumthangkha
Tshangla
Manange
Thakali
Western Tamang
Kaike
Rongpo
Bunan
Darma
Byangsi
Pattani
Kinnauri
Toto
Dhimal
Bangru
Anu-Hkongso
Nung (Myanmar)
Drung
Raute
Nyishi-Hill Miri
Tagin
Galo
Bokar-Ramo
Apatani
Tangam
Milang
Lahta-Zayein Karen
Kayan Lahwi
S'gaw Karen
Geba Karen
Eastern Kayah
Tawra
Enu
Mpi
Honi
Hani
Akha
Cosao
Bisu
Youle Jinuo
Lahu Shi
Lahu
Nusu
Sani
Axi Yi
Azha
Wusa Nasu
Wuding-Luquan Yi
Sichuan Yi
Lolopo
Lipo
Lisu
Xishanba Lalo
Katso
Lashi
Chashan
Zaiwa
Longchuan Achang
Pela
Maru
Burmese
Ugong
Situ
Japhug
Khroskyabs
Southern Qiang
Northern Qiang
Southern Pumi
Northern Pumi
Zhaba
Narua
Naxi
Shixing
Guiqiong
Ersu
Dimasa
Bodo-Mech
Rabha
Atong (India)
Garo
Northern Jinghpaw
Southern Jinghpaw
Kado
Chak
Konyak Naga
Chang Naga
Nocte Naga
Mandarin Chinese
Wutunhua
Tangwang
Wu Chinese
Min Nan Chinese
Duhumbi
Southern Bai
Central Bai
Northern Bai
Northern Tujia
Kman

Fig. S5. The facing trees of the Sino-Tibetan family with values of nominal case (on the left) and verb-final word order (on the right). Red stands for the presence of the grammatical feature, while gray indicates its absence. Both case and verb-final word order are absent in the languages of the Karenic branch (Lahta-Zayein Karen, Kayan Lahwi, S'gaw Karen, Geba Karen, and Eastern Kayan), whereas other clades possess both of these features.

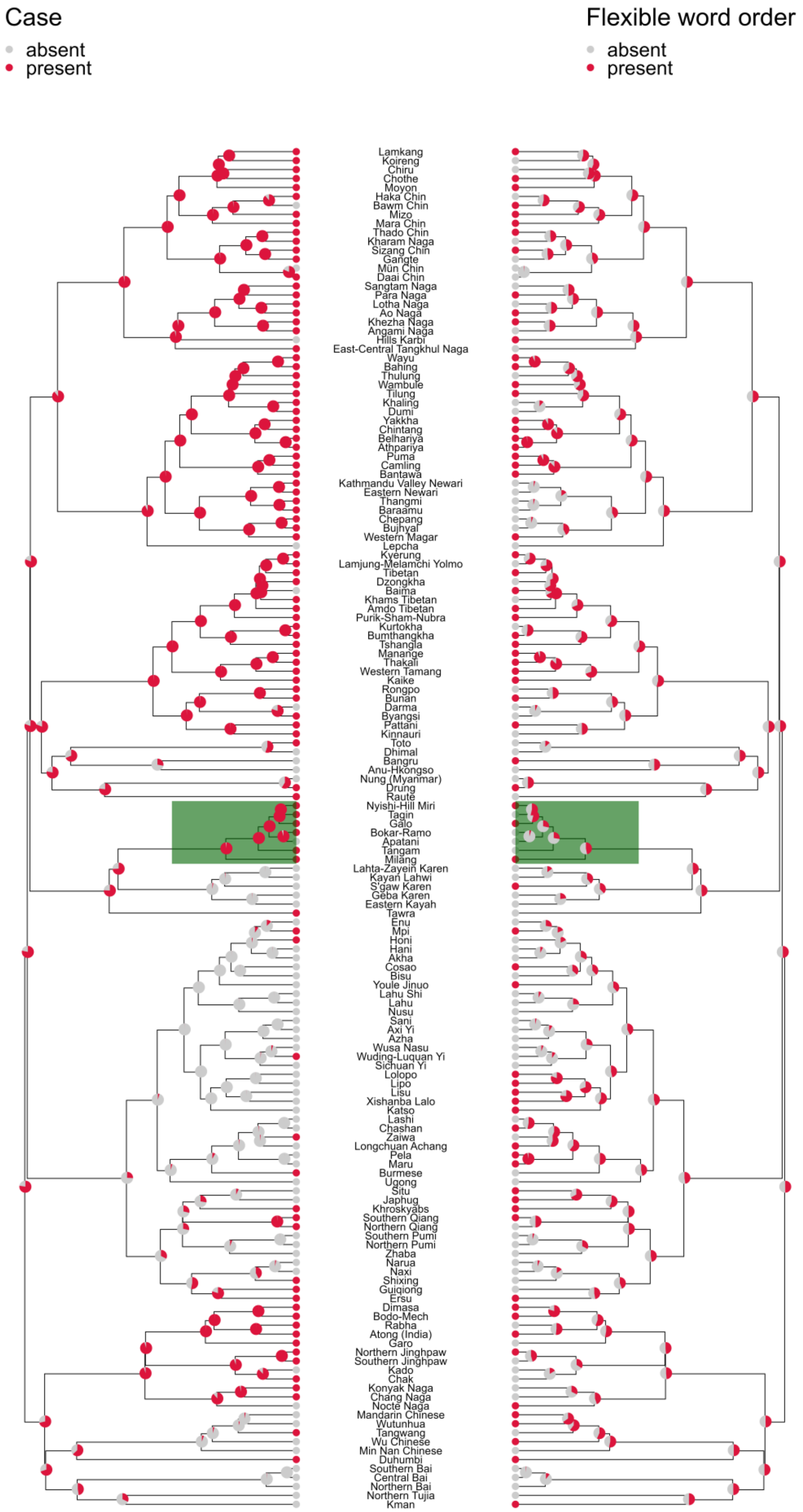
Case
absent
present
Flexible word order
absent
present
Lamkang
Koireng
Chiru
Chothe
Moyon
Haka Chin
Bawm Chin
Mizo
Mara Chin
Thado Chin
Kharam Naga
Sizang Chin
Gangte
Mün Chin
Daai Chin
Sangtam Naga
Para Naga
Lotha Naga
Ao Naga
Khezha Naga
Angami Naga
Hills Karbi
East-Central Tangkhul Naga
Wayu
Bahing
Thulung
Wambule
Tilung
Khaling
Dumi
Yakkha
Chintang
Belhariya
Athpariya
Puma
Camling
Bantawa
Kathmandu Valley Newari
Eastern Newari
Thangmi
Baraamu
Chepang
Bujhyal
Western Magar
Lepcha
Kyerung
Lamjung-Melamchi Yolmo
Tibetan
Dzongkha
Baima
Khams Tibetan
Amdo Tibetan
Purik-Sham-Nubra
Kurtokha
Bumthangkha
Tshangla
Manange
Thakali
Western Tamang
Kaike
Rongpo
Bunan
Darma
Byangsi
Pattani
Kinnauri
Toto
Dhimal
Bangru
Anu-Hkongso
Nung (Myanmar)
Drung
Raute
Nyishi-Hill Miri
Tagin
Galo
Bokar-Ramo
Apatani
Tangam
Milang
Lahta-Zayein Karen
Kayan Lahwi
S'gaw Karen
Geba Karen
Eastern Kayah
Tawra
Enu
Mpi
Honi
Hani
Akha
Cosao
Bisu
Youle Jinuo
Lahu Shi
Lahu
Nusu
Sani
Axi Yi
Azha
Wusa Nasu
Wuding-Luquan Yi
Sichuan Yi
Lolopo
Lipo
Lisu
Xishanba Lalo
Katso
Lashi
Chashan
Zaiwa
Longchuan Achang
Pela
Maru
Burmese
Ugong
Situ
Japhug
Khroskyabs
Southern Qiang
Northern Qiang
Southern Pumi
Northern Pumi
Zhaba
Narua
Naxi
Shixing
Guiqiong
Ersu
Dimasa
Bodo-Mech
Rabha
Atong (India)
Garo
Northern Jinghpaw
Southern Jinghpaw
Kado
Chak
Konyak Naga
Chang Naga
Nocte Naga
Mandarin Chinese
Wutunhua
Tangwang
Wu Chinese
Min Nan Chinese
Duhumbi
Southern Bai
Central Bai
Northern Bai
Northern Tujia
Kman

Fig. S6. The facing trees of the Sino-Tibetan family with values of nominal case (on the left) and flexible word order (on the right). Red stands for the presence of the grammatical feature, while gray indicates its absence. Almost all languages from the Macro-Tani branch in our sample (except for Apatani) possess case, and some of these languages, such as Nyishi-Hill Miri, Galo, and Milang have likely developed flexible word order.

Table S1. The results of *K*-fold cross-validation of three models fit with *brms*, which incorporate random effects and predict the distribution of case, verb-final word order and flexible word order.

Model	Response variable	ΔELPD	ΔSE
spatial	Case	0.00	0.00
phylogenetic		-0.67	0.69
spatial + phylogenetic		-1.25	0.67
spatial	Verb-final word order	0.00	0.00
spatial + phylogenetic		-0.07	0.59
phylogenetic		-0.17	0.68
spatial + phylogenetic	Flexible word order	0.00	0.00
spatial		-0.33	0.44
phylogenetic		-1.19	0.61

Table S2. The coefficients of the fixed effects (with the 95% credible intervals) in three best-supported models (*b*, *c*, and *d*) by Phylogenetic Path Analysis fit with *brms* to test the robustness of the results.

Response	Predictor	Model	Estimate	Est.Error	Q2.5	Q97.5
Flexible word order	Case	model b	0.33	0.18	-0.02	0.68
Verb-final	Case		1.76	0.59	0.67	3.01
Flexible word order	Case	model c	0.34	0.18	-0.01	0.68
Case	Verb-final		1.49	0.33	0.86	2.15
Verb-final	Case	model d	1.75	0.59	0.66	2.99
Case	Flexible word order		0.25	0.22	-0.18	0.68
